# Supplementary material for: The allometric growth relationship between electricity consumption and economics in China
Source: PLoS One. 2023 Nov 15;18(11):e0291671. doi: 10.1371/journal.pone.0291671 (PMC10651011; doi:10.1371/journal.pone.0291671)
Supplement: Attachment — Submitted filename: Response to Reviewers.docx [file pone.0291671.s003.docx]

Dear reviewer,

We are very grateful for your affirmation of our manuscript and your valuable comments to us. These comments are very valuable and helpful to the revision and improvement of our manuscript. We have carefully studied your comments and completed the correction. We hope it will be approved. The main corrections in the paper and responses to your comments are as follows:

(Original comments are in italic, we reply to the question highlighted with red color, the added context and significant change in revised manuscript highlighted with green in paper.)

Reviewer #1: All comments have been addressed, but some minor problems still exist as follows:

***Point 1:*** Syntax error and title consistency issue on line11-13: --study the relationship between electricity consumption and allometric economic growth in China at multiple scales..--

Response 1: Thank you for your suggestion. According to your suggestion, we have made the following modification:

line 12-13 --study the allometric growth relationship between electricity consumption and economics in China at multiple scales.--

***Point 2:*** There is one blank cell should be filled on top line in Table2 .

**Response 2:** Thank you very much for your suggestion. According to your suggestion, we have filled in the blank in Table 2:

line 387-388 **Table 2**. Scale coefficients of electricity consumption and GDP abnormal growth at different stagesof economic development in the eight economic zones

| Regions | 1972-1978 | 1979-2003 | 2004-2017 |
| --- | --- | --- | --- |
| Northeast region | 1.0215 | 2.7375 | 2.1534 |
| Northern coast | 0.7148 | 2.128 | 1.5209 |
| Eastern coast | 0.8614 | 1.918 | 1.5649 |
| Southern coast | 0.3877 | 1.5045 | 1.6328 |
| Middle reaches of the Yellow River | 0.5685 | 1.9769 | 1.5343 |
| Middle reaches of Yangtze River | 0.5356 | 2.1687 | 1.67 |
| Southwest region | 0.8229 | 1.7864 | 1.6132 |
| Northwest region | 0.5839 | 1.8207 | 1.0687 |

***Point 3:*** In Fig.4, different colors stand for conomic zones, but it is not clear in the figure.

**Response 3:** Thank you for pointing out the issue about the figure. According to your suggestion, we have made the following modification:

line 354-355

|  |  |
| --- | --- |
|  |  |

Fig.4 Relationship between electricity consumption and GDP growth in the three economic zones

Reviewer #2: The authors have improved the paper according to the reviewers' comments, this paper can be accepted after making the following minor revisions.

***Point 1:*** The title needs to be modified to: The Allometric Growth Relationship Between Electricity Consumption and Economics in China.

**Response 1:** Thank you for your suggestion. According to your suggestion, we have revised the title to: The Allometric Growth Relationship Between Electricity Consumption and Economics in China.

***Point 2:*** Some minor errors in the abstract need to be corrected.

**Response 2:** Thank you for pointing out the error in my article. We have checked and revised the content of the abstract, and the revised content is as follows:

line 6-31 Abstract: Electric power is the basic industry of a modern country. The rapid development of the power industry has promoted economic development and social progress. With the establishment of the carbon neutrality target of carbon peak, China’s power industry is also facing new situations and new challenges. This paper innovatively introduces the concept of allometric growth in biology, and uses the data of provincial electricity consumption and economic development from 1972 to 2017, to study the allometric growth relationship between electricity consumption and economics in China at multiple scales. Combined with the spatial autocorrelation analysis method, the temporal and spatial characteristics of allometric growth between electricity consumption and economic development were researched based on the analysis of spatial correlation of GDP electricity consumption intensity. The results show that: (1) The provincial GDP electricity consumption intensity shows an aggregate distribution, and the local aggregate pattern changed significantly from 1972 to 2017. (2) The High-High Cluster gradually disappeared, and the Low-Low Cluster moved southward, appearing in the middle and lower reaches of the Yangtze River. (3) The differential growth coefficient of electricity consumption and economic development increased initially and decreased from 1972 to 2017. (4) GDP would increase by 7.4%, 20.1%, and 15.6% in the simulation of electricity consumption, increasing 10% at the three stages of China's economic development. (5) The allometric growth coefficients of electricity consumption and economic development are very different in the three economic belts, eight economic zones, and provinces with different characteristics at the different stages of economic development. Formulating differentiated regulation policies for regional economic development and electricity consumption will be conducive to the coordinated development of electric power production and the national economy.

***Point 3:*** The introduction of methods should precede the introduction of data.

**Response 3:** Thank you for your suggestion. According to your suggestion, we have adjusted the order in which methods and data are introduced.

***Point 4:*** The title of the third section should be Results, and the sub-headings of the third section are too long and should be more concise.

**Response 4:** Thank you very much for your valuable comments. We have changed the title of the third section to Results, and simplified some of the sub-headings in the third section.

## line 326-328 3.2 The Allometric Growth Relationship Between Electricity Consumption and Economics

### 3.2.1 China

### line 356 3.2.2 Three Economic Belts

### line 390 3.2.3 Eight Economic Zones

### line 419 3.2.4 Province (City)

***Point 5:*** The title of the fourth part should be Discussion, the authors should focus on describing the differences between the article study and other scholars' studies, thus highlighting the relevance and academic value of the article. This literature should be helpful for your research: Compilation of Water Resource Balance Sheets under Unified Accounting of Water Quantity and Quality, a Case Study of Hubei Province.

**Response 5:** Thank you for your suggestion. According to your suggestion, we have changed the title of the fourth section to Discussion. In this section,we have quoted the article you recommended and described the differences between the article study and other scholars' studies. The revised content is as follows:

line 452 4. Discussion

## line 463-467 In this paper, the allometric growth relationship between electricity consumption and economics in China has been studied at multiple scales. In contrast, some articles from the perspective of water resources improved the value accounting method for water resources by assessing water efficiency and quality [37].

line 723-726 37. Liang Yuan; Liwen Ding; Weijun He; Yang Kong; Thomas Stephen Ramsey; Dagmawi Mulugeta Degefu; Xia Wu. Compilation of Water Resource Balance Sheets under Unified Accounting of Water Quantity and Quality, a Case Study of Hubei Province[J]. Water,2023,Vol.15(1383): 1383

***Point 6:*** The conclusion part of the paper should give a brief introduction of the research background and methods, and then introduce the main conclusions.

**Response 6:** Thank you very much for your valuable comments. A brief introduction of the research background and methods has been included in the concluding section of the paper.

line 552-557 Electricity development is closely linked to economic and social development. In this article, combined with the spatial autocorrelation analysis method, the temporal and spatial characteristics of allometric growth between electricity consumption and economic development were researched based on the analysis of the spatial correlation of GDP electricity consumption intensity. The conclusions are as follows:

***Point 7:*** The format of the full text and references should be standardized.

**Response 7:** Thank you for your suggestion. We have adjusted the format of the full text and references.

line 28 the different stages of economic development. Formulating differentiated regulation

line 46 and people's life [3]

line 47 supply [4]

line 636-637 6. Yoo S H. Electricity consumption and economic growth: evidence from Korea[J]. Energy Policy, 2006, 1(1/4):235-243.

...

Yi Qi, Mengyuan Tang, Dongsheng Dang and Caijuan Qi

Aug. 24, 2023
